# The clinical utility of intraoperative blink reflex monitoring and its synergistic value with lateral spreading response monitoring in predicting postoperative outcomes in patients with hemifacial spasm following microvascular decompression

**DOI:** 10.1080/07853890.2026.2700159

**Published:** 2026-07-13

**Authors:** Jun Yang, Xiaocui Yang, Jiajia Liu, Ke Li, Jiawei Shi, Yingzhun Liang, Shuangshuang Liang, Hanjie Liu, Hui Qiao, Xing Fan

**Affiliations:** a Beijing Neurosurgical Institute, Capital Medical University, Beijing, China; b Beijing Tiantan Hospital, Capital Medical University, Beijing, China

**Keywords:** Hemifacial spasm, microvascular decompression, blink reflex, lateral spread response

## Abstract

**Background:**

Microvascular decompression (MVD) is the current definitive treatment for achieving a radical cure of primary hemifacial spasm (pHFS). The current study aimed to integrate blink reflex (BR) monitoring into MVD for pHFS to assess surgical efficacy and explore its predictive value for postoperative spasm when combining with lateral spread response (LSR) monitoring.

**Methods:**

A prospective cohort study included 105 patients with pHFS undergoing MVD. Intraoperative zygomatic LSR (ZYG-LSR), mandibular LSR (MAN-LSR), and BR were monitored. Multivariate logistic regression was used to assess independent predictors of postoperative spasm at 3 months after surgery. Two predictive models (Model 1: LSR; Model 2: LSR plus BR) for postoperative spasm were compared using Receiver Operating Characteristic (ROC) curve analysis at four postoperative time points: 1 day, 1 week, 1 month, and 3 months, with the area under the curve (AUC) quantifying overall accuracy and the DeLong test for comparison.

**Results:**

BR showed a higher elicitation rate (99.0%) than ZYG-LSR (97.1%) and MAN-LSR (91.4%). Persistent ZYG-LSR (odds ratio 28.99), MAN-LSR (odds ratio 12.06), and BR (odds ratio 10.38) were identified as independent predictors for postoperative spasm at 3 months after surgery (all *p* < 0.05). Regarding the two predictive models, their performance improved over time. At 3 months, Model 2 showed a numerically higher AUC than Model 1 (0.955 versus 0.901), while the DeLong test still did not reach statistical significance (*p* = 0.083).

**Conclusion:**

In conclusion, intraoperative BR monitoring appears to be a feasible and potentially useful adjunct to conventional LSR monitoring in MVD for pHFS, and warrants further evaluation in larger studies.

## Introduction

Hemifacial spasm (HFS) is a prevalent chronic neuromuscular disorder characterized by involuntary, paroxysmal, and progressively worsening contractions of facial muscles on the affected side. It typically begins in middle age or later, and demonstrates a clear female predominance. Epidemiological data indicate that the global prevalence of HFS is approximately 14.5 per 100,000 for women and 7.4 per 100,000 for men, resulting in millions of individuals worldwide being affected [1–3]. Although not life-threatening, persistent spasms often lead to social discomfort and psychological distress, thereby leading to HFS gaining clinical attention from neurologists and neurosurgeons.

Primary HFS (pHFS) accounts for over 90% of HFS cases, with the core pathogenic mechanism linked to vascular compression of the facial nerve root exit zone, most commonly by the posterior or anterior inferior cerebellar artery [4]. Two major pathophysiological hypotheses have been proposed to explain the progression of the disease. The central hypothesis posits that vascular compression induces hyperexcitability of the facial nucleus. In contrast, the peripheral hypothesis posits that chronic mechanical compression leads to demyelination of facial nerve axons, resulting in aberrant ephaptic transmission between adjacent nerve fibres [5,6]. Regardless of the specific mechanism, the fundamental therapeutic principle for pHFS is the alleviation of vascular compression at the facial nerve. Consequently, surgical intervention, particularly microvascular decompression (MVD), is currently considered the definitive treatment for achieving a radical cure in patients with primary hemifacial spasm [7].

Intraoperative neurophysiological monitoring (IONM) plays a critical role in MVD for pHFS. For instance, brainstem auditory evoked potential (BAEP) monitoring is utilized to preserve auditory nerve function, while electromyography (EMG) is employed to protect the facial nerve. Both modalities serve as essential safety measures during surgery, consistent with the primary objective of most intraoperative monitoring techniques [8,9]. In contrast, the lateral spread response (LSR), defined as an abnormal muscle response elicited by stimulation of one branch of the facial nerve and recorded from a non-target ipsilateral branch, serves a distinct purpose. The intraoperative disappearance of LSR is utilized as an indicator of adequate vascular decompression, with the primary objective of assessing surgical efficacy rather than merely preventing complications. Numerous previous studies have investigated the predictive value of LSR for postoperative outcomes in patients with pHFS undergoing MVD, with inconsistent findings [9–11]. Our recent work has validated the clinical significance of two-branch LSR monitoring, that is, zygomatic LSR (ZYG-LSR) and mandibular LSR monitoring, in predicting postoperative spasm [12]. However, according to our research findings, even with two-branch LSR monitoring, discrepancies between LSR findings and clinical outcomes persist in some patients, suggesting that current LSR-based efficacy monitoring has its limitations and requires refinement.

To improve the accuracy of intraoperative efficacy monitoring during MVD, two primary strategies can be proposed. One is the adoption of multi-branch LSR monitoring instead of two-branch LSR monitoring, thereby enhancing the spatial assessment of facial nerve hyperexcitability [13]. The other approach is to integrate novel techniques to establish a multimodal monitoring framework, enabling a more comprehensive evaluation. The blink reflex (BR), an electrophysiological response mediated by the trigeminal and facial nerves, has been suggested as a potential indicator of sufficient decompression during MVD [14,15]. However, its clinical utility remains limited due to a lack of supporting evidence. In this study, we aimed to integrate BR monitoring into MVD for patients with pHFS and explore its predictive value for surgical outcomes when combined with two-branch LSR monitoring.

## Methods

### Patient population

A prospective cohort study was conducted on patients with pHFS who underwent MVD in the Department of Neurosurgery, Beijing Tiantan Hospital, Capital Medical University, between September 2023 and August 2024. The diagnosis of pHFS was confirmed based on typical clinical manifestations and neuroimaging examinations to exclude secondary causes, such as intracranial tumours or vascular malformations. Inclusion criteria were defined as follows: (1) age over 18 years with a confirmed diagnosis of pHFS; (2) preoperative facial EMG documenting the presence of LSR, with no evidence of other neuromuscular disorders; (3) with no prior neurosurgical intervention for HFS; (4) willingness to participate. Exclusion criteria included: (1) incomplete demographic or clinical data; (2) failure to elicit LSR and BR intraoperatively; (3) loss to follow-up or failure to complete at least 3 months of follow-up.

A total of 108 consecutive patients were initially screened, and three were excluded due to intraoperative failure to record LSR and BR, resulting in a final sample size of 105 patients. Demographic and clinical data were extracted from electronic medical records. Intraoperative electrophysiological data (ZYG-LSR, MAN-LSR, BR) were retrieved from the institutional database. Follow-up data were collected through clinic visits and telephone interviews on 1 day, 1 week, 1 month, and 3 months postoperatively. This study was approved by the Ethics Committee of Beijing Tiantan Hospital, and written informed consent was obtained from all patients or their legal representatives before surgery. The study was conducted in accordance with the principles outlined in the Declaration of Helsinki.

### Anaesthesia and surgical procedures

All patients received combined intravenous and inhalational anaesthesia, administered by the same team to ensure consistency. Anaesthesia induction was achieved with propofol, sufentanil, and atracurium. For anaesthesia maintenance, a continuous infusion of propofol and remifentanil was used, combined with sevoflurane at a minimum alveolar concentration of ≤ 0.5. No muscle relaxants were administered during the surgical procedure except for induction.

All surgical procedures were performed by the same neurosurgical team using a standardized retrosigmoid approach. Patients were positioned in the lateral decubitus position, and a postauricular curvilinear incision was made, followed by craniotomy to expose the cerebellopontine angle. Upon dural opening, cerebrospinal fluid was gradually drained to reduce intracranial pressure and optimize visualization of the operative field. The responsible vessel compressing the facial nerve root was identified under high-resolution microsurgical visualization and was gently separated from the facial nerve. A Teflon pad was interposed between the vessel and the nerve to achieve stable decompression. Adequate decompression was initially defined by the disappearance of intraoperative abnormal LSR and BR waveforms. In cases where these waveforms persisted after primary decompression, the entire cisternal portion of the facial nerve was carefully re-evaluated for residual vascular compression, with supplementary decompression performed for any identifiable compression that could be safely addressed. If LSR or BR waveforms remained despite comprehensive exploration and intervention, the procedure was concluded to minimize the risk of potential iatrogenic injury. Following confirmation of adequate decompression by the surgeon, the dura mater and incision were closed.

### Intraoperative electrophysiological monitoring

IONM was performed using the Cadwell Cascade Elite System (Cadwell Industries, Kennewick, WA, USA), with twisted-pair subdermal needle electrodes for LSR and BR monitoring. All procedures were conducted by a neurophysiologist with at least 5 years of relevant experience, in close coordination with the surgical team. The monitoring was performed according to the protocols described in our previous studies [12,16].

For ZYG-LSR monitoring, the anode was placed perpendicularly at the midpoint between the tragus and lateral canthus, and the cathode was positioned 2 cm medially. The active recording electrode was over the mentalis muscle, with the reference 2 cm lateral. For MAN-LSR monitoring, the anode was placed along the mandibular margin, with the cathode 2 cm lateral to it. The active recording electrode was on the orbicularis oculi beneath the pupil, and the reference was 2 cm lateral. Constant-current stimulation was delivered (0.3 ms pulse duration, 1.1 Hz frequency, 10–30 mA intensity). Signals were band-pass filtered at 10–1500 Hz. For BR monitoring, stimulation was applied at the supraorbital notch on the affected side, with recordings from the orbicularis oculi beneath the pupil. Each stimulus comprised four biphasic constant-current pulses (200 μs pulse width, 2 ms interval, 10–30 mA intensity). Responses were averaged from two consecutive stimuli of reversed polarity, using 100–1500 Hz band-pass filtering. The R1 component of BR was applied for further analysis.

Baseline waveforms were recorded after cerebrospinal fluid release. Routine recordings were obtained every 2–5 min, and at least once per minute during critical steps. Responses were immediately compared with the baseline and communicated to the surgical team. Patients who failed to elicit both LSR and BR were excluded according to predefined criteria.

### Statistical analysis

Statistical analysis was performed using SPSS Statistics (Version 24.0, IBM Corp., Armonk, New York, USA). Categorical variables are reported as frequencies and percentages, whereas continuous variables are summarized using medians and interquartile ranges (IQR). Group comparisons were performed using the Chi-square test, Fisher’s exact test, Student’s t-test, or nonparametric tests as appropriate to screen for variables associated with postoperative spasm. Multivariate binary logistic regression was applied to assess the predictive value of intraoperative monitoring parameters for patient outcomes, specifically postoperative spasm at three months. The odds ratio (OR) and the corresponding 95% confidence interval (CI) were reported to indicate variable significance.

Finally, two binary logistic regression models were constructed to predict postoperative outcomes. Model 1 included ZYG-LSR and MAN-LSR as binary predictors (0 = negative, 1 = positive). Model 2 additionally incorporated BR as a third binary predictor. Receiver operating characteristic (ROC) curve analysis was used to evaluate the predictive performance of the models, with the area under the curve (AUC) quantifying overall accuracy. The difference in predictive accuracy between the two models was evaluated using the DeLong test. A p-value of < 0.05 was considered statistically significant. Data visualization was conducted using R software (Version 4.3.1; R Foundation for Statistical Computing, Vienna, Austria) with the “pROC” and “ggplot2” packages.

## Results

### Patient characteristics

The demographic and clinical characteristics of the 105 patients enrolled in the study are presented in Table 1. The cohort showed a female predominance (67 cases, 63.8%), with a median age of 53 years (IQR: 47–59 years). At 3 months postoperatively, 13 patients (12.4%) still presented with facial spasm, while 92 patients (87.6%) achieved complete symptom relief. A comparative analysis of demographic and clinical characteristics between the postoperative spasm group and the non-spasm group revealed no significant differences (*p* > 0.05 for all). These characteristics include age, gender, height, weight, affected side, disease duration, history of botulinum toxin injection and acupuncture, comorbidities (hypertension and diabetes), intraoperative blood loss, and classification of incision.

**Table 1.** Univariate analysis of patient characteristics associated with postoperative spasm at three months.

Variables		All (*n* = 105)	No Spasm (*n* = 92)	Spasm (*n* = 13)	P
Age, median [IQR]		53 [47.000,59.000]	53 [47.000,60.000]	51 [47.000,58.000]	0.347
Gender, N (%)	female	67 (63.810)	57 (61.957)	10 (76.923)	0.293
	male	38 (36.190)	35 (38.043)	3 (23.077)	
Height, median [IQR]		164 [160.000,170.000]	165 [160.000,170.000]	160 [158.000,170.000]	0.339
Weight, median [IQR]		65 [59.000,75.000]	65 [60.000,75.000]	61 [55.000,71.000]	0.352
Affected Side, N (%)	left	51 (48.571)	44 (47.826)	7 (53.846)	0.684
	right	54 (51.429)	48 (52.174)	6 (46.154)	
Disease Duration, median [IQR]		4 [2.000,6.000]	4 [2.000,5.000]	6 [4.000,8.000]	0.056
Botulinum Toxin, N (%)	no	93 (88.571)	81 (88.043)	12 (92.308)	0.651
	yes	12 (11.429)	11 (11.957)	1 (7.692)	
Acupuncture, N (%)	no	68 (64.762)	60 (65.217)	8 (61.538)	0.795
	yes	37 (35.238)	32 (34.783)	5 (38.462)	
Hypertension, N (%)	no	72 (68.571)	62 (67.391)	10 (76.923)	0.488
	yes	33 (31.429)	30 (32.609)	3 (23.077)	
Diabetes, N (%)	no	100 (95.238)	89 (96.739)	11 (84.615)	0.055
	yes	5 (4.762)	3 (3.261)	2 (15.385)	
Intraoperative Blood Loss, median [IQR]		50 [50.000,50.000]	50 [50.000,50.000]	50 [50.000,50.000]	0.472
Surgical Incision, N (%)	I	57 (54.286)	49 (53.261)	8 (61.538)	0.575
	II	48 (45.714)	43 (46.739)	5 (38.462)	
ZYG-LSR, N (%)	negative	80 (78.431)	77 (86.517)	3 (23.077)	<0.001
	positive	22 (21.569)	12 (13.483)	10 (76.923)	
MAN-LSR, N (%)	negative	85 (88.542)	81 (94.186)	4 (40.000)	<0.001
	positive	11 (11.458)	5 (5.814)	6 (60.000)	
Blink Reflex, N (%)	negative	70 (67.308)	67 (73.626)	3 (23.077)	0.001
	positive	34 (32.692)	24 (26.374)	10 (76.923)	

### Predictive value of IONM indicators for postoperative spasm

**Figure 1.**
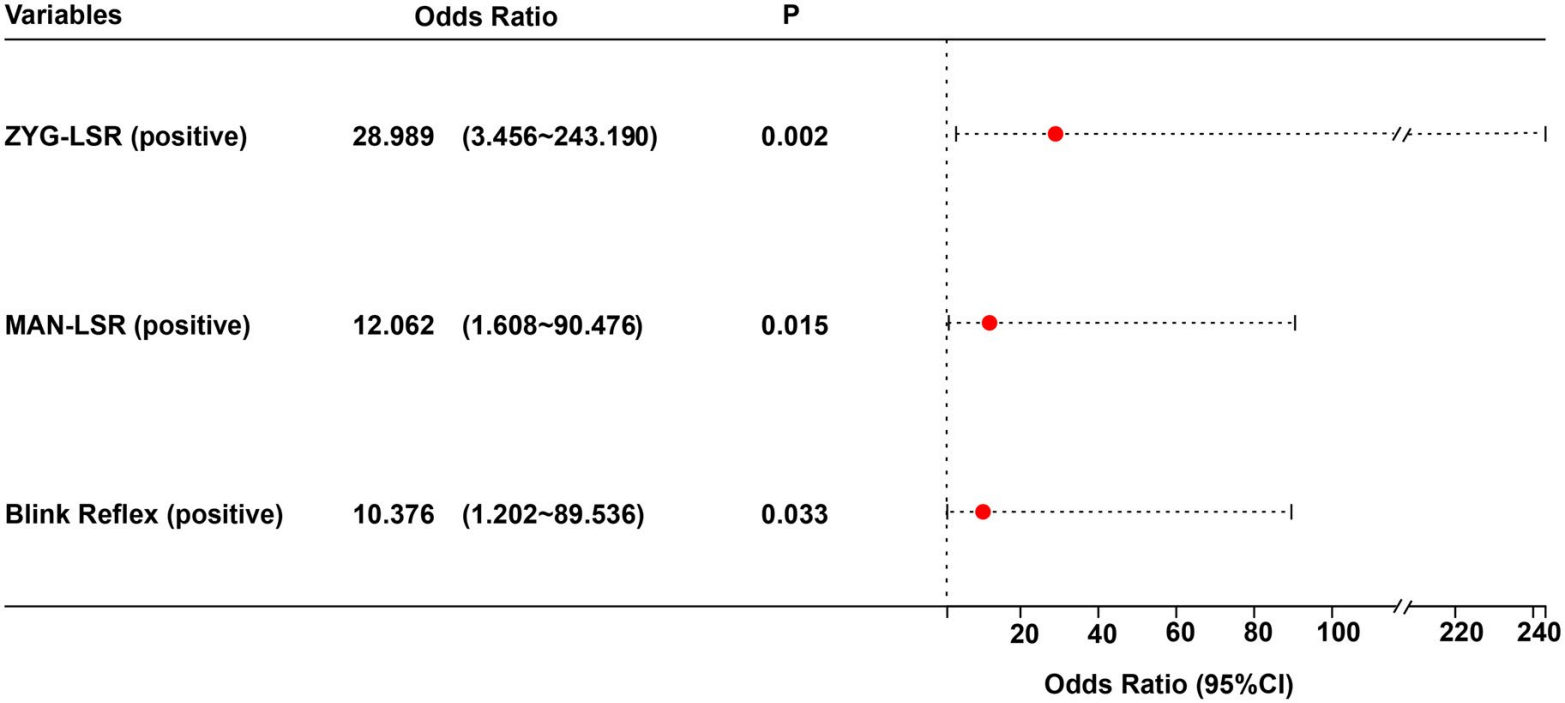
Forest plot of multivariate logistic regression analysis for independent predictors of postoperative spasm at 3 months. The plot illustrates the odds ratios (ORs) and 95% confidence intervals (CIs) of three intraoperative electrophysiological indicators: zygomatic lateral spread response (ZYG-LSR), mandibular LSR (MAN-LSR), and blink reflex (BR). All three indicators were confirmed as independent predictors of postoperative spasm.

The elicitation rates of ZYG-LSR, MAN-LSR, and BR in the study cohort were 97.1% (102/105), 91.4% (96/105), and 99.0% (104/105), respectively. BR exhibited synchronized changes with LSR before and after decompression, and a representative case is presented in Supplementary Figure S1. The detailed temporal relationship between intraoperative BR disappearance and specific surgical steps is provided in Supplementary Figure S2. Univariate analysis revealed statistically significant differences in the presence of these intraoperative neurophysiological monitoring parameters between patients with and without postoperative spasm at 3 months (*p* < 0.001 for LSRs; *p* = 0.001 for BR; Table 1). Specifically, the persistence rates of ZYG-LSR, MAN-LSR, and BR in the spasm group were 76.9%, 60.0%, and 76.9%, respectively, which were significantly higher than those in the non-spasm group (13.5%, 5.8%, and 26.4%, respectively). Subsequently, multivariate logistic regression analysis confirmed that persistent ZYG-LSR (OR 28.989, 95% CI 3.456–243.190, *p* = 0.002), MAN-LSR (OR 12.062, 95% CI 1.608–90.476, *p* = 0.015), and BR (OR 10.376, 95% CI 1.202–89.536, *p* = 0.033) were all independent predictors of postoperative spasm at 3 months (Figure 1).

### Temporal performance of IONM-based predictive models

**Figure 2.**
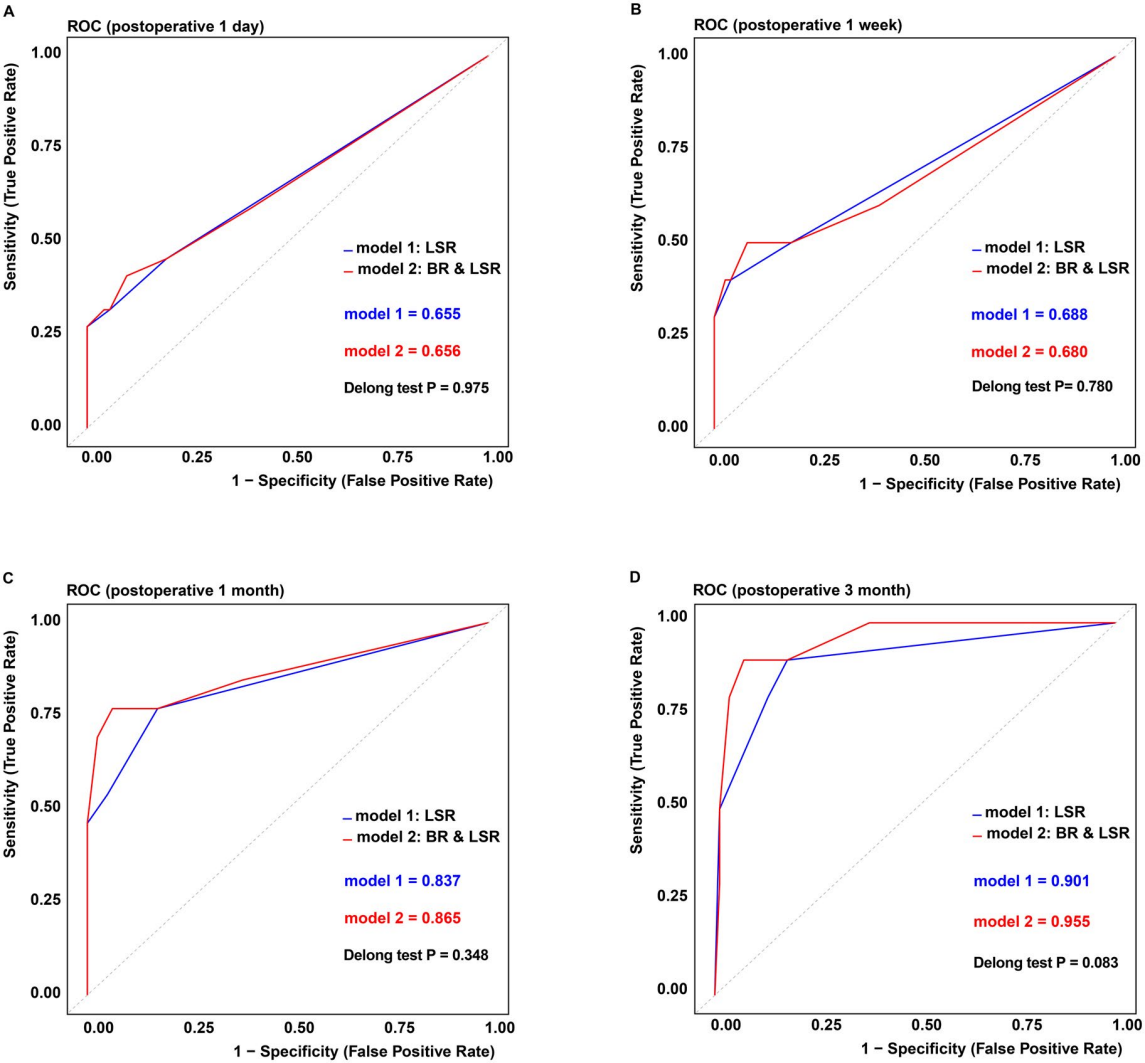
Receiver operating characteristic (ROC) curves comparing the predictive performance of two models at four postoperative time points. Model 1 was constructed based solely on zygomatic lateral spread response (ZYG-LSR) and mandibular LSR (MAN-LSR), while Model 2 integrated ZYG-LSR, MAN-LSR, and blink reflex (BR). (A) Postoperative day 1; (B) Postoperative week 1; (C) Postoperative month 1; (D) Postoperative month 3.

During the follow-up period (from postoperative day 1 to 3 months), 17 patients showed delayed relief (25 patients with spasm at day 1), and five patients suffered recurrent spasms. Two predictive models were constructed to evaluate postoperative spasm using data from 93 patients, all of whom had elicitable ZYG-LSR, MAN-LSR, and BR. Model 1 was based solely on LSR monitoring, and Model 2 was based on the combination of LSR and BR monitoring. The predictive performance of both models showed a consistent upward trend over time, with significant improvements from the early postoperative period to 3 months postoperatively (Figure 2).

On postoperative day 1, the AUC values were 0.655 for Model 1 and 0.656 for Model 2, with a Delong test p-value of 0.975 (Figure 2(A)). By postoperative week 1, the AUC values were slightly adjusted to 0.688 (Model 1) and 0.680 (Model 2), and the *p*-value decreased to 0.780 (Figure 2(B)). At 1 month postoperatively, the AUC values of both models increased numerically, while the DeLong p-value decreased to 0.156, which remained non-significant (Figure 2(C)). At 3 months postoperatively, Model 2 achieved the highest AUC of 0.955, compared with 0.901 for Model 1, and the DeLong test p-value dropped to 0.083 (Figure 2(D)). Detailed AUC values (with corresponding 95% confidence intervals) and DeLong test results at each postoperative time point are provided in Supplementary Table 1. Although the difference between the two models did not reach statistical significance at any time point, the numerical decrease in DeLong p-values and the numerical increase in AUC difference towards 3 months should therefore be regarded as an exploratory trend.

## Discussion

The present study prospectively enrolled 105 patients with pHFS undergoing MVD and investigated the clinical utility of BR monitoring during the surgical procedure. The results showed that persistent BR was an independent predictor of postoperative spasm, and the multimodal model integrating LSR and BR demonstrated progressively greater predictive accuracy over the single LSR model as neural function recovery advanced. The findings confirmed that BR monitoring served as an effective adjunct to LSR monitoring in assessing MVD efficacy, and further underscored the clinical significance of multimodal neurophysiological monitoring for optimizing intraoperative strategies and improving postoperative prognostic accuracy.

The blink reflex is a polysynaptic trigemino-facial reflex evoked by supraorbital nerve stimulation and recorded from the orbicularis oculi muscle. It consists of an early ipsilateral R1 component with mediated by pontine oligosynaptic pathways, and a later bilateral R2 component relayed through the medulla and reticular formation with longer latency and greater variability. Conventionally, BR serves as a tool to evaluate the integrity and excitability of trigemino-facial pathways and brainstem circuits in neurological disorders such as facial neuropathy, brainstem lesions, and relevant movement disorders, but its intraoperative application was historically limited due to technical constraints associated with general anaesthesia [17,18]. Reliable recording of the R1 component under anaesthesia was first demonstrated by the pioneering work of Deletis et al. which established the foundation for intraoperative BR monitoring [19]. Subsequently, their team also conducted valuable investigations into BR monitoring during MVD for pHFS [20]. In 2020, Choi et al. first reported the clinical significance of BR monitoring in MVD for pHFS, highlighting its potential as an indicator of adequate decompression [14]. Our team has prior experience with BR monitoring in vestibular schwannoma resection [16]. Upon extending BR monitoring to MVD, we were initially surprised to find that its functional role in these two surgical contexts differed significantly. In vestibular schwannoma resection, BR is a physiological signal reflecting the integrity of the trigeminal-facial pathway, and IONM aims to preserve it to prevent neural injury. In MVD, BR is a pathological response indicating persistent compression and neural hyperexcitability, and IONM targets its disappearance to confirm effective decompression. Moreover, according to our clinical observations, BR could not be elicited intraoperatively on the contralateral healthy side of pHFS patients. This finding further supports that intraoperative BR represents a pathological response rather than a normal physiological reflex in pHFS patients. The absence of BR on the unaffected side may result from abnormal hyperexcitability within the trigeminal-facial neural pathway of pHFS patients, leading to an elevated response threshold of the entire circuit [21,22]. These observations highlight the unique role of BR monitoring in MVD for pHFS, distinguishing it from applications in other neurosurgical procedures, even including MVD for trigeminal neuralgia [23].

The current study provides preliminary evidence supporting the potential clinical utility of BR monitoring in MVD for pHFS. First, BR exhibited a higher elicitation rate compared to ZYG-LSR and MAN-LSR under intraoperative conditions. This high feasibility supports the technical applicability of BR monitoring in this surgical setting. Second, multivariate logistic regression analysis confirmed that persistent BR is an independent predictor of postoperative spasm, alongside ZYG-LSR and MAN-LSR. This finding confirms that BR monitoring provides unique prognostic information beyond that obtained from LSR alone, serving as a valuable adjunct to conventional LSR monitoring. Third, ROC curve analysis revealed a numerical, exploratory trend in predictive performance. The multimodal model incorporating LSR and BR showed numerically higher AUC values than the LSR-only model at later postoperative time points, although the AUC differences did not reach statistical significance at any time point by DeLong testing. Given that neural function recovery following MVD is a dynamic process, the combined model may potentially capture additional aspects of pathological resolution in pHFS; however, this hypothesis remains exploratory and requires confirmation in larger studies. Cumulatively, these findings suggest the potential value of BR monitoring in MVD for pHFS, supporting its consideration as part of multimodal intraoperative monitoring.

Moreover, we also investigated the timing distribution of BR disappearance, and found that BR disappearance rarely occurred immediately during Teflon pad placement but most frequently presented at the final stage after the decompression manoeuvre. This finding suggests that BR is more appropriately regarded as a final intraoperative efficacy marker rather than a real-time indicator of decompression. Clinically, neurosurgeons do not need to overemphasize dynamic BR changes during the decompression procedure. Instead, it is advisable that neurosurgeons can complete Teflon pad placement just according to their microsurgical experience, and then allow an observation window of 5–10 min to monitor whether BR disappears. This practical strategy may help standardize and optimize the clinical application of intraoperative BR monitoring in MVD.

Notably, the complementary value of BR to LSR may arise from their different physiological mechanisms. LSR primarily reflects abnormal ephaptic transmission between facial nerve branches resulting from demyelination at the root exit zone due to neurovascular compression, whereas BR engages the trigeminal-facial reflex pathway and brainstem interneuronal circuits [15]. The abnormalities in pHFS potentially reflect both peripheral ephaptic effects and secondary central nervous system hyperexcitability or plasticity induced by chronic vascular compression. The combined model, integrating BR and LSR monitoring, can cover both peripheral nerve conduction and trigeminal-facial network function, enabling a more comprehensive evaluation of decompression adequacy, which may account for its numerically higher predictive performance.

Our findings are consistent with the prior study of Choi et al. which first highlighted the potential role of BR monitoring in MVD for pHFS, and extend this work by quantifying the independent predictive value of BR monitoring and demonstrating its synergistic benefit with LSR monitoring over time [14]. Most prior studies on the IONM of MVD for pHFS have primarily focused on LSR monitoring, with limited integration of additional IONM modalities aimed explicitly at assessing surgical efficacy [24–26]. By establishing a practical, multimodal monitoring framework, our study provides a clinically actionable approach to enhancing intraoperative monitoring efficacy and improving postoperative prognostic accuracy.

This study has several limitations that should be acknowledged. First, its single-center prospective design and small sample size may limit the generalizability of the findings and increase the risk of statistical bias. Second, BR is inherently a physiological trigemino‑facial reflex. Intraoperative BR changes may be influenced by several non‑specific factors, including fluctuations in anaesthetic depth, minor variations in stimulation or recording conditions, and mild mechanical manipulation of the facial nerve pathway during surgery. These factors should be considered when interpreting BR as a pathological response specific to pHFS. Finally, the present study focused on the qualitative presence or absence of LSR and BR, without quantifying waveform parameters such as amplitude or latency, which may provide additional prognostic information. Future multicenter studies with larger sample sizes and extended follow-up are warranted to address these limitations.

## Conclusion

BR exhibits high intraoperative elicitation feasibility, serves as an independent predictor of postoperative spasm, and yields numerically higher predictive performance when combined with LSR compared to LSR alone. Overall, intraoperative BR monitoring is a reliable and valuable adjunct to LSR monitoring, and it may be considered for multimodal monitoring in MVD for pHFS.

## Supplementary Material

Figure S1.tif

Table S1.docx

Figure S2.tif

## Data Availability

The data that support the findings of this study are available on request from the corresponding author. The data are not publicly available due to privacy or ethical restrictions.
