# Supplementary figures and images for: The clinical utility of intraoperative blink reflex monitoring and its synergistic value with lateral spreading response monitoring in predicting postoperative outcomes in patients with hemifacial spasm following microvascular decompression
Source: Ann Med. 2026 Jul 13;58(1):2700159. doi: 10.1080/07853890.2026.2700159 (PMC13366649; doi:10.1080/07853890.2026.2700159)

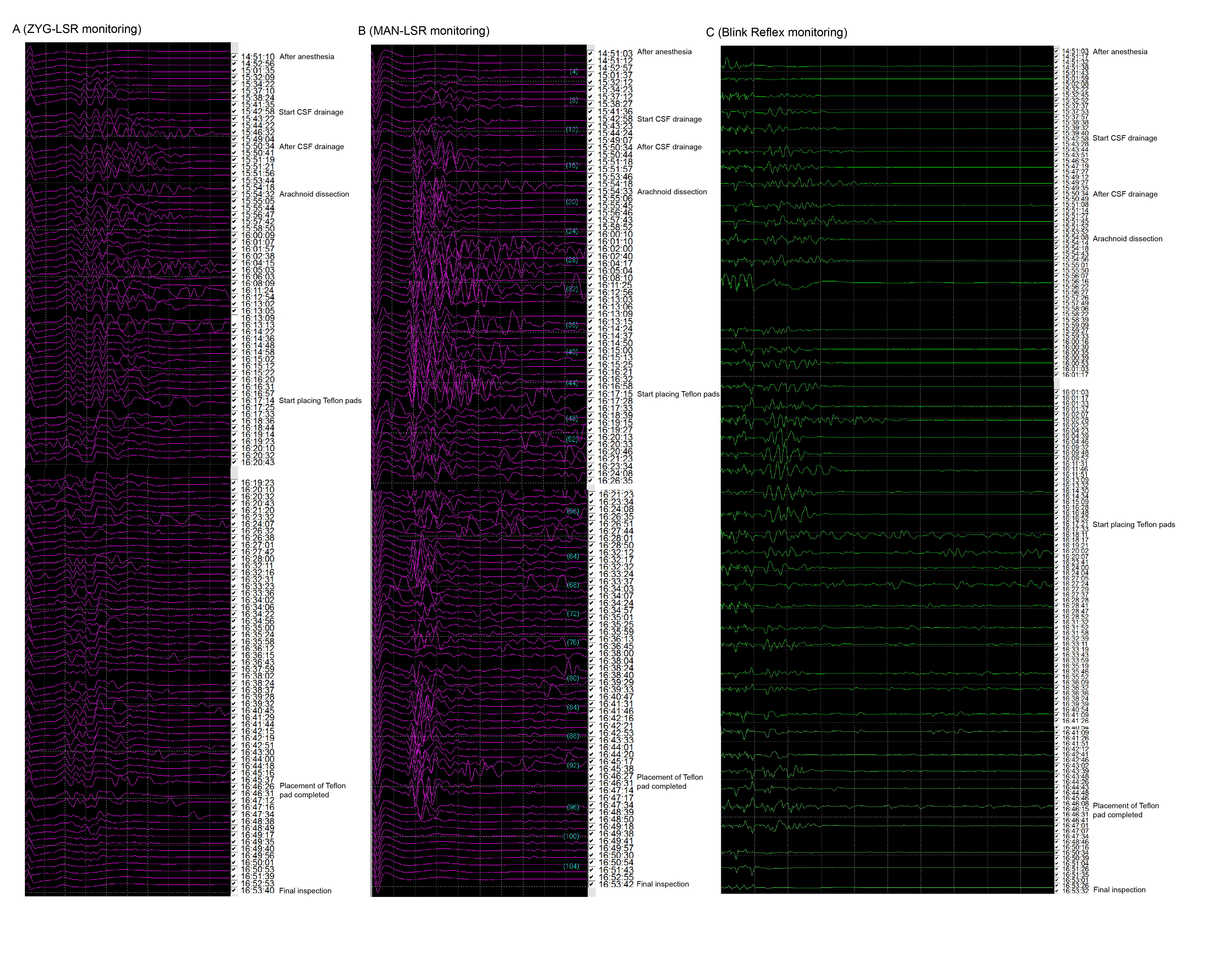

Supplement: Figure S1.tif [file IANN_A_2700159_SM1545.tif]

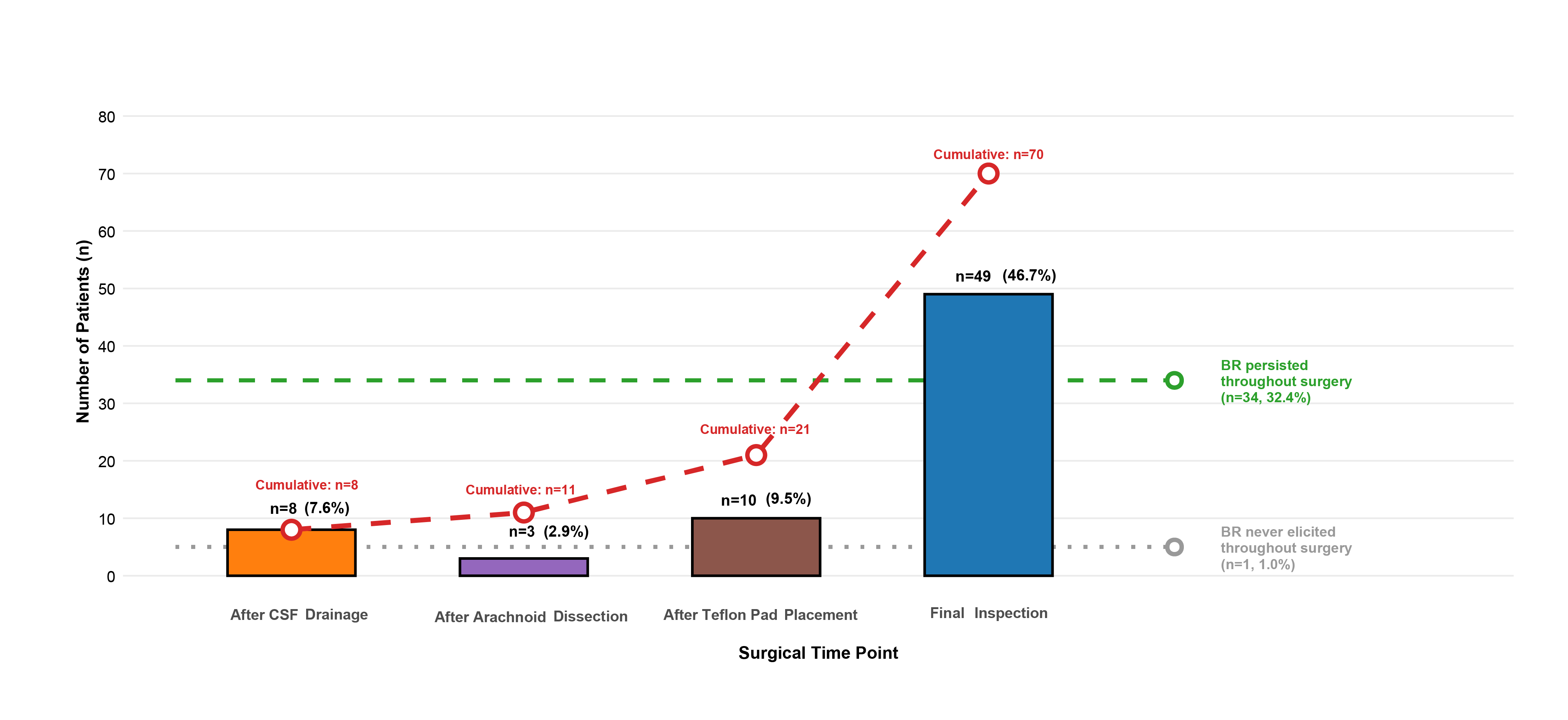

Supplement: Figure S2.tif [file IANN_A_2700159_SM1543.tif]
