# Supplementary material for: The clinical utility of intraoperative blink reflex monitoring and its synergistic value with lateral spreading response monitoring in predicting postoperative outcomes in patients with hemifacial spasm following microvascular decompression
Source: Ann Med. 2026 Jul 13;58(1):2700159. doi: 10.1080/07853890.2026.2700159 (PMC13366649; doi:10.1080/07853890.2026.2700159)
Supplement: Table S1.docx [file IANN_A_2700159_SM1544.docx]

| Time | Model 1 AUC (95% CI) | Model 2 AUC (95% CI) | DeLong p |
| --- | --- | --- | --- |
| POD 1 | 0.655 (0.530-0.780) | 0.656 (0.516-0.796) | 0.975 |
| POW 1 | 0.688 (0.556-0.820) | 0.680 (0.530-0.831) | 0.78 |
| POM 1 | 0.887 (0.701-0.972) | 0.901 (0.723-1.000) | 0.156 |
| POM 3 | 0.901 (0.786-1.000) | 0.955 (0.897-1.000) | 0.083 |

*(POD = postoperative day 1; POW = postoperative week 1; POM = postoperative month 1.)*
